# A Qualitative Approach to Extract Diagnostic Patterns of Cognitive Impairment in Parkinson’s Disease

**DOI:** 10.21203/rs.3.rs-7329834/v1

**Published:** 2025-12-03

**Authors:** A. Journey Eubank, Abhilash Thatikala, Maryam Y. Garza, Humaira Khan, Tuhin Virmani, Rohit Dhall, John Talburt, Linda J. Larson-Prior, Fred W. Prior

**Affiliations:** University of Arkansas for Medical Sciences; University of Arkansas for Medical Sciences; The University of Texas Health Science Center at San Antonio; University of Arkansas for Medical Sciences; University of Arkansas for Medical Sciences; University of Arkansas for Medical Sciences; University of Arkansas at Little Rock; University of Arkansas for Medical Sciences; University of Arkansas for Medical Sciences

**Keywords:** Clinical reasoning, Parkinson’s disease, Cognitive impairment, Montreal Cognitive Assessment, Qualitative research, Biomedical informatics

## Abstract

**Background:**

Parkinson’s disease (PD) is the second-most diagnosed age-related neurodegenerative disorder globally. PD pathology causes dysregulation of motor movement and for many, mild or minor cognitive impairment (PD-MCI). The most recommended global screening exam to detect PD-MCI is the Montreal Cognitive Assessment© (MoCA). Traditionally, the MoCA is scored according to guidelines and compared against a standardized cutoff, but clinical professionals additionally draw upon their observations of the patient’s performance to determine the score. To better understand how clinicians use the MoCA in real-world clinical settings, we employed the qualitative descriptive approach to identify performance patterns professionals utilize to assess the cognitive health of a person with PD.

**Methods:**

We curated retrospective data from nine people with PD-MCI to PD-Dementia. Each patient had one completed MoCA exam and one neuropsychological report containing health data. The assessments were organized into three groups of three and used in semi-structured interviews with six clinical professionals to gather at minimum two clinical opinions for each.

**Results:**

Three coders distilled, based on consensus, three clinically meaningful patterns from the interviews composed of features emphasized as vital by the interviewees for determining a person’s cognitive health. The derived features were from a patient’s performance on sections of the MoCA exam, sociodemographic and health data from the neuropsychological report, and dependent relationships between the assessments.

**Conclusions:**

Our study leveraged the popular MoCA exam to explore its real-world clinical use. Extracting these patterns clinicians recognized provides deeper insights into how they interpret cognitive health creating a blueprint for future efforts to tailor the exam for detecting cognitive impairment in people with PD.

## Background

Parkinson’s disease (PD) is a complex neurodegenerative disorder that leads to motor deficits, cognitive impairments, and neuropsychiatric symptoms, such as apathy and depression.[1, 2] While dysregulation of movement in PD is well characterized, cognitive impairment in PD presents a unique diagnostic challenge, ranging from mild or minor (PD-MCI)[3] to major cognitive impairment (PD-Dementia)[4] as defined by the Movement Disorder Society. PD-MCI is a distinct intermediate state between PD with normal cognition and PD-Dementia. The presentations of cognitive impairment and disease progression can vary between individuals, but two cognitive subtypes are frequently recognized in the literature: one characterized by frontostriatal and executive dysfunction, often resulting in bradyphrenia, and another involving posterior cortical deficits, marked by visuospatial problems and language/semantic fluency impairments.[5] Rather than a fixed stage, PD-MCI should be thought of as a spectrum: some people remain at the PD-MCI stage for a long time, some can revert to normal cognition for a period, while others progress to dementia.[6, 7] Notably, those who revert to normal cognition after a diagnosis of PD-MCI have an elevated risk of declining back to PD-MCI within the next year.[7]

This cognitive variability is particularly relevant given that PD-MCI is identified in 20—50% of people at the time of initial PD diagnosis,[1, 8] increasing to 40—57% after approximately 5 years of disease duration.[6, 9–11] These trends underscore the importance of research focused on understanding the presentations, or patterns, of cognitive impairment in PD. The wide variability in PD-MCI prevalence at diagnoses may, in part, stem from differences in the cognitive domains assessed and psychometric properties of the exams used. This suggests a need for assessments specifically calibrated for PD-MCI. Improving the sensitivity and specificity of these tools could also aid in earlier identification of cognitive impairment in people with PD so therapeutic interventions have a greater chance of potentially slowing disease progression.

The Movement Disorder Society provided guidelines for testing cognitive impairment in PD, including Level II neuropsychological exam batteries—the gold standard for diagnosis—and Level I rapid global screening exams (e.g., the Montreal Cognitive Assessment© [MoCA]).[12–14] Currently, when it comes to Level II diagnostic gold standards, clinical practice often faces access barriers, such as the lack of available neuropsychologists, time, cost, or a patient’s lack of insurance coverage.[3] Therefore, the Level I rapid global cognitive exams are most commonly used to gauge cognitive health.[3, 15, 16] The MoCA is the most widely recommended rapid global screening exam, but debates on the accuracy of the exam’s scoring method and its specificity and sensitivity for detecting PD-MCI are ongoing.[17, 18] According to the MoCA guidelines, the total score is calculated by summing the points from each scorable section, with each cognitive domain weighted based on its assigned point value, and adding an additional point for individuals with ≤ 12 years of education.[19] The summation of the section scores is then compared to a standardized predetermined scale with ≥ 26 out of 30 indicating normal cognition. Building on this, discussions with clinicians revealed that, rather than relying solely on the MoCA total score compared to a cutoff as the determinant of cognitive health, they instead interpret patterns in a patient’s performance in the context of health-related data. These health-related contextual dependencies are of particular interest, as understanding the patterns that clinicians identify as representative of PD-MCI could lay the groundwork for developing a new method of interpreting the MoCA that improves the sensitivity and specificity for people with PD.

Using semi-structured interviews and the “think aloud protocol” [20, 21], we investigated the clinical applications of the MoCA. We sought to extract, concurrently, the clinically meaningful features of patient performance on the MoCA exam and health-related data that clinicians consider vital in determining the presence of cognitive impairment in people with PD. By distilling clinically meaningful features, we aimed to synthesize diagnostically significant patterns across the cognitive impairment spectrum in PD.

## Methods

### Study Design

This study employed a descriptive qualitative methodology to explore the perspectives of a specific population.[22] Guided by the pragmatist’s paradigm, which emphasizes real-world observation through the context of a relevant but subjective source, we used purposive sampling to focus on insights from clinical neurologists and neuropsychologists specializing in movement disorders in adult and geriatric populations.[23] The Standards for Reporting Qualitative Research checklist was used to ensure transparent reporting of this study (**Supplemental 1**).[24] This research approved by the University of Arkansas for Medical Science’s Institutional Review Board (IRB Number 274288). All procedures involving human participants followed ethical guidelines in accordance with the Declaration of Helsinki and its later amendments.

### Reflexivity of Researchers

The interdisciplinary research team on this project provided a diverse range of expertise to guide this research and provide valuable insights into real-world clinical practice. Three experienced neurologists provided the foundational concepts and domain-specific expertise critical to the study’s conceptualization, and provided continued input throughout the project (TV, RD, HK). An experienced qualitative researcher (MYG) gave valuable guidance on qualitative methodology and trained the lead investigator in interviewing techniques. The lead investigator, (AJE, a biomedical informatics PhD candidate), an expert biomedical informaticist (FWP), and a neurology resident (AT) collaborated to extract the categories and codes to formulate the patterns from the interviews. Including a neurology resident to assist in distilling information from the interviews provided an additional valuable perspective and ensured the informaticists interpreted insights from the interviews accurately and extracted features vital to constructing clinically relevant patterns. An experienced neuroscientist and researcher (LJL-P) lent her extensive expertise in the field of PD research, offering excellent perspectives on cognitive impairment in PD. A researcher in the field of information quality (JT) provided valuable expertise on data quality practices in research to ensure rigorous and transparent data reporting.

### Description of Participants

The Arkansas Clinical Data Repository team at the University of Arkansas for Medical Sciences curated and deidentified completed MoCA exams and neuropsychological reports from the electronic health record system, Epic, in accordance with regulatory requirements.[25] The curation criteria for the retrospective patient data for this study were: (i) Primary diagnosis of PD and a secondary diagnosis of MCI or Dementia from PD in their medical record; (ii) A completed MoCA exam and neuropsychological report in the health record.

During the study design, it was determined that a total of nine patients’ MoCA exams and neuropsychological reports were optimal based on the availability of recruited clinical professionals and the number of assessments that could be reasonably reviewed in a 60-minute interview time constraint. The nine MoCA exams and neuropsychological reports were identified with the assistance from clinical colleagues in the Neurology and Geriatric Departments at the University of Arkansas for Medical Sciences. The assessments were organized into three groups of three sets of exam-report pairs (herein referred to as patient profiles). The neuropsychological reports contained a comprehensive overview of the patient, describing presenting concerns, biopsychosocial history, psychiatric symptoms, activities of daily living, neurobehavioral status, cognitive domain test results, clinical impressions, diagnostic summary, and recommendations from the time of the neuropsychological appointment. Notably, while the health information was current at the neuropsychological appointment, the completed MoCA exam in their file could have been administered prior to or after the time of the neuropsychological appointment date. Table 1 provides the administration date for both assessments and the final diagnosis by patient ID number. The assessments were completed with a mean difference of 1.70 years (SD ≈ 1.38 years). Despite this time gap, the report generated from the neuropsychological appointment was a vital assessment that succinctly provided pertinent health information traditionally available if the interviewee were the patient’s clinical specialist, thereby simulating real-world clinical practice. In our retrospective data set, three patients were diagnosed with PD with mild cognitive impairment nonamnestic (PD-MCI non-A), two were categorized as amnestic mild cognitive impairment (PD-MCI A), and two were classified as PD-Dementia (Table 1).

All potential interviewees were provided a brief overview of the research purpose and participant protections at the time of recruitment to review upon deciding to participate, thus acknowledging they understood the benefits and risks of participation. A total of six clinical professionals were recruited, clinical neurologists (n = 5) and a neuropsychologist (n = 1), from two different hospital systems to participate in the interviews and ensure we gathered, at minimum, two opinions on each patient profile. The recruitment criteria required potential participants to be currently practicing clinical neurologists or neuropsychologists specializing in movement disorders in the adult to geriatric population. The advising neurologist (RD), the senior author and biomedical informatics expert (FWP), and the lead investigator (AJE) recruited potential interviewees by corresponding with colleagues, associates, and mentors.

### Data Collection

Verbal informed consent was obtained from all volunteer interviewees prior to beginning the interview recording. The interviewees received their three assigned patient profiles one week in advance and were asked to review them prior to the interview. The semi-structured interviews were formatted using the think aloud protocol,[21] an interview technique designed to study thought processes, which has previously been leveraged to gain insights into medical decision-making for diagnostic techniques and investigations into the clinical understanding of cognitive impairment.[26–28] The think aloud protocol requires the interviewee to speak their thoughts aloud as they perform a specific task, thereby providing a verbal account of their critical thinking and decision-making processes.

The interviewer (AJE) utilized an interview guide developed by research team members (AJE, FWP, RD, MYG) (**Supplemental 2**). The interview questions were developed using an iterative approach and pilot-tested with an advising clinician (RD) to determine applicability. The purpose of the interview guide was to assist the interviewer in encouraging the interviewees to articulate their observations as they reviewed their three assigned patient profiles. The questions focused on the patterns of patient performance on the MoCA exam, what important health features an interviewee would want to know from the neuropsychological report, and how they interpreted any context dependence between the two assessments and if they agreed with the diagnosis of the patient. For example, the question, “What do you observe/look for in the patient’s performance?” was used in the MoCA section to prompt detailed descriptions of clinicians’ observation and interpretive practices when reviewing the exam. A similar question was used in the neuropsychology report portion of the guide: “What do you look for in this section? What does its presence/absence mean to you?” To continually prompt the interviewee to verbalize their thought processes as they reviewed the documents this question was used: “Could you talk me through the aspects of the narrative that helped you determine that?” Additionally, the guide functioned as a reference for the interviewer to keep the discussion focused and within the allotted time frame. To further simulate real-time clinical practice procedures, the interviewees were instructed to share their computer screens during the interview, allowing them to review the patient profiles freely according to their workflow methodology.

A trained qualitative researcher (AJE) conducted interviews via videoconference lasting ≤ 60 minutes. The interviews were recorded and stored on a HIPAA-compliant platform. All interviews were transcribed verbatim by AJE. To ensure interviewee privacy protections, personal identifiers in the transcripts were deidentified using an alphanumeric code prior to sharing with colleagues contributing to the interview coding process.

### Data Processing and Analysis

Recurrent themes in the data were identified by inductive and deductive hybrid thematic analysis.[29, 30] The adaptive approach allowed inductive elements to emerge by identifying key features essential to the differential diagnostic process. Simultaneously, deductive reasoning ensured a focus on the research question: whether the features noted as important for determining cognitive health by the interviewees formed diagnostically meaningful patterns of cognitive impairment in PD. As this study was largely explorative, features of importance were extracted by recursive open coding of the transcripts,[31] which allowed for refinement and triangulation as the interviews progressed to enhance the rigor of the deductive and inductive content analysis and subsequent extraction of patterns.[31, 32]

Table 2 displays an example of the methods used to derive important features underlying the codes and categories from interview quotes that reflecting clinicians’ observations. The table shows the document the interviewee was discussing while making an observation about patient performance on the MoCA, relationships between MoCA performance and health-related data from the neuropsychological report, and other clinically relevant details from the report that may impact MoCA performance.

The data was input into Microsoft Excel, using a row per patient profile (n = 9), grouping features of importance from the assigned interviewees to establish one pattern per patient profile. Features were deemed important based on collaborative discussions between AJE and FWP, as they reviewed the interview transcripts creating codes and categories according to their interpretations of what the interviewee pointed out as diagnostically meaningful. A neurology resident (AT) was recruited to review the interview transcripts independently, extract features of importance and organize these features into patterns for each patient profile as AJE and FWP had previously done. Meetings between the three interview coders (AJE, FWP, AT) were held to reach a consensus on each patient profile’s important features and respective pattern. Then, all three interview coders independently reviewed each pattern to determine if any could be combined using these devised pattern reduction rules: (i) are there clinically meaningful differences and, (ii) can the features be quantified? Clinical meaningfulness was based on the frequency a specific feature was mentioned in an interview and its co-occurrence with other features. Only features that could be quantitively represented—either using a binary indicator for presence (1) or absence (0), or numerically (e.g., structural similarity index of the cube example and patient drawing from the MoCA)—were retained. A consensus meeting was held to finalize the list of patterns.

To statistically compare patient profile demographics and cognitive exam scores between the three independent groups (PD-MCI non-A, PD-MCI A, PD-Dementia) the Kruskal-Wallis test was used. This test is preferred for small sample sizes—requiring only two people per group—and it does not assume normality or equal variance, which is appropriate given our unequal and small group sizes.

## Results

Table 3 displays the patient demographics and the Kruskal-Wallis statistical test effect sizes for the patient’s age, number of years of education, total MoCA score and the Repeatable Battery for the Assessment of Neuropsychological Status (RBANS)[33] total index score. The nine patient profiles were divided into three groups according to the diagnostic impression in the neuropsychological report (PD-MCI non-A, PD-MCI A, PD-Dementia). The Kruskal-Wallis Test revealed a significant difference in RBANS scores across diagnostic groups (H (2) = 6.11, *p* = .047, η^2^ = 0.76). Post-hoc Dunn’s test with Bonferroni correction showed that the PD-MCI non-A group scored significantly higher than the PD-Dementia group (adjusted *p*-value = 0.043), which is expected as a higher score suggests better cognitive functioning.

During an interview, it was discovered that the performance of patient ID 6 on the MoCA exam and cognitive assessment battery in the neuropsychological report was most consistent with a diagnosis of delirium rather than impairment due to PD. This resulted in removal of this patient profile, as it could not be definitively determined that the patient’s performance on the MoCA exam was unaffected by delirium, thereby reducing the number of patient profiles to eight (n = 8). Patient ID 1 was added to the affected group of three patient profiles to supplement the deficit, resulting in three clinical opinions for this patient profile. Additionally, one of the two clinicians that reviewed Patient ID 8 expressed that they disagreed with the diagnosis of PD-MCI non-A recorded in the neuropsychological assessment report. Instead, they suggested that the patient’s performance on the MoCA and extent of affected activities of daily living recorded in the neuropsychological report reflected mild PD-Dementia.

Table 2 illustrates how codes and categories were derived to capture specific features that clinicians considered necessary for their differential diagnostic processes as they reviewed the patient profiles. The ‘Important Feature(s)’ column presents the synthesized features from interview quotes, reflecting how clinicians integrated medical history (e.g., medication lists), contextual health features that influence MoCA performance (e.g., hearing issues), and broader personal information (e.g., presence of family support) to interpret the patient’s cognitive functioning beyond the MoCA total score. An example of how we used qualitative methods to synthesize the important features composing the patterns represented in Table 4 can be seen by directing your attention to the third quote in Table 2. This quote shows how a clinical professional utilized contextual patient health information to interpret performance on the MoCA. The quote is extracted from an interview where the clinician was reviewing the Memory subtask of the MoCA exam and noted that when the patient was supposed to repeat the word ‘face’ instead presumably said ‘faith’, per a note on the exam document. This note prompted the interviewee to consider if the patient had a hearing impairment. This important feature is reflected in Pattern 3 of Table 4 in the ‘MoCA Section’ column noted as ‘Memory: number of tries, correlate to hearing issue’.

Several features were repeatedly identified by the majority of interviewees as clinically relevant to interpreting a patient’s performance on the MoCA or important contextual health information. Consequently, these features appeared in all three final patterns and are recorded in the ‘Recurrent Features’ row of Table 4. The repetition reflects consistent clinical relevance based on the patter reduction criteria described above, demonstrating that pattern features are not mutually exclusive.

In the ‘MoCA Section’ column of Table 4, there are several correlations mentioned between a specific cognitive domain section and health-related data from the neuropsychological report. The Language and Attention feature in Pattern 1 of the ‘MoCA Section’ column, includes checking performance for lexical fluency. Lexical capabilities are often diminished when cognitive functions are degraded by Alzheimer’s disease pathology, cardiovascular issues,[34] or vascular risk factors,[35] making these critical contextual health features to check for and are noted within the ‘Health Data’ column. In Pattern 2, a feature in the Attention and Language domain sections in the ‘MoCA Section’ column notes that task performance must be normalized by education. This feature points to the importance of contextual biopsychosocial factors, as individuals with higher educational degrees tend to perform better on these tasks.[36] In Pattern 3, the Memory feature listed in the ‘MoCA Section’ column illustrates how a clinician might suspect that a physical health-related feature is impacting exam performance. To complete the Memory subsection of the MoCA exam, patients must listen to words said by the exam administrator and repeat them back; however, if a patient has hearing aids or a hearing impairment and performs poorly, the results may reflect a physical limitation rather than a cognitive dysfunction.

Similarly, in the Clock task of the ‘MoCA Section’ within Patterns 2 and 3, one feature notes the small size of the analog clock numbers, referred to in the literature as ‘micrographia’, a sequela of PD commonly linked to motor dysfunction.[37] While this may appear irrelevant for understanding presentations of the cognitive impairment spectrum in PD, patterns are produced by the extraction of features of importance that clinical professionals recognized while interpreting the assessments. The patterns in Table 4 offer a holistic perspective of patient health derived from the eight model patient profiles used to create them. Therefore, we did not eliminate features involving motor symptoms as they were determined to be clinically meaningful because they were verbalized by the clinical professionals.

## Discussion

Our objective was to distill diagnostically meaningful patterns of cognitive impairment in people with PD by examining how clinicians interpreted the MoCA in conjunction with patient health data. The interviews with clinical experts revealed the discrepancy between the currently recommended MoCA scoring method and its clinical application. Each section of the MoCA exam includes a checkbox for task completion and the exam instructions advise the proctor to award or deduct points based on specific criteria. For example, giving a point if the patient drew relatively parallel lines for reproducing a 3D cube in the Cube sub-section, or deduct a point for an incorrect word recall in the Delayed Recall section. This research supported the insights shared by clinical mentors during the initial conceptualization of the study, that clinicians move beyond adherence to the MoCA scoring instructions. Instead, relying on pattern recognition—an integral part of differential diagnostics—to interpret patterns of patient performance on the MoCA exam in relation to health features. Our results, summarized in Table 4 illustrate these important features that clinicians see as diagnostically vital, such as noting the presence of arthritis—noted in Pattern 3—which could impact their drawing of a 3D cube, or if the patient is taking medications that could impair memory capacity—noted in Pattern 2.[30, 38] This is particularly notable given that medications used to treat the motor and non-motor symptoms in PD can cause side effects like sleep disturbances, drowsiness, or memory problems.[39] Additionally, many people with PD experience anxiety, depression, and apathy, which can affect their performance on global screening exams like the MoCA, further highlighting the importance of integrating contextual health information into exam scoring procedures.[40] These relationships are typically considered implicitly by clinicians during exam interpretation; however, our analysis makes this diagnostic reasoning explicit.

Other rapid screening tools with comparable administration time to the MoCA exam such as the Parkinson’s Disease-Cognitive Rating Scale,[41] the Scales for Outcomes of Parkinson’s Disease,[42] or the Parkinson Neuropsychometric Dementia Assessment[43] are specifically designed for detecting cognitive deficits in people with PD but are not commonly employed in the clinical setting. The assimilation of new tools in the clinic is often lengthy and challenging, as evidenced by the high failure rate of clinical decision support systems.[44] This difficulty is primarily due to the disruptive impact on clinical workflows, compounded by the need for training and certifications necessary for successful implementation. Following this evidence, a similar challenge would likely arise with the introduction of a new global screening tool, which may help explain the reason why PD-specific screening exams are not as widely used as the MoCA. This reasoning prompted the selection of the MoCA exam as the model global screening tool for this study. Additionally, we believe that optimizing an established and popular global screening exam would provide the foundation for a more rapid acceptance of any future recommended scoring alterations.

Our research has several limitations that must be acknowledged. The first is the small sample size of both interviewees (n = 6) and the number of patient profiles (n = 8). The small sample size of interviewees was a result of limited access to clinical neurologists and neuropsychologists in the home institution the study was conducted. Five interviewees were recruited from the study’s home institution, and one interviewee was recruited from an external healthcare institution. Our cohort had five clinical neurologists, who are considered experts in their respective fields of neurology and one neuropsychologist who sees many patients with movement disorders. Recruitment of only one neuropsychologist was due to limited availability of these specialized professionals. Particularly those who frequently work with older adults with movement disorders. Additionally, it must be mentioned that this study’s premise is to explore patterns of patient performance on the MoCA in relation to their health data. Neuropsychologists are experts in curating cognitive batteries specifically tailored for analysis of discrete aspects of a patient’s cognition and do not traditionally utilize the MoCA exam as a component of their battery. For this reason, while prudent to include a neuropsychologist’s opinion given their insights into the neuropsychological effects of a neurodegenerative disorder such as PD, it was not necessary to recruit additional participants. Furthermore, inductive thematic saturation was achieved during analysis of the interview transcripts as we used purposive sampling to gather a homogenous expert population of clinical professionals specializing in movement disorders and geriatrics. The small sample size of patient profiles (n = 9) was based on maximizing the number of patient assessments that could be reasonably reviewed within the 60-minute interview time limit leading to the to the conclusion that reviewing three patient profiles per interview would be practical.

Another limitation is that the final number of patterns (n = 3) is small. However, these patterns emerged through thematic saturation based on the recurrence of specific features across all interviews and the frequency with which certain features appeared together. Arguably, a larger number of patterns could be extracted by decomposing these three patterns, but focusing on a smaller set of distilled patterns offers a more targeted approach to identifying whether these patterns can be meaningfully detected in a larger dataset. This focused approach enhances our ability to explore how clinical professionals interpret patient performance on the MoCA and health-related data when mining for differential diagnostic insights. We also would like to acknowledge that one of the primary purposes of the MoCA is to track longitudinal changes in cognition. The exam’s inclusion of a broad range of cognitive domains, especially visuospatial executive functions which are commonly affected by PD progression, makes it a valuable screening tool. However, for the purposes of this study, a single time point was sufficient to capture the important features clinicians note during their differential diagnostic processes.

An additional limitation was the time frame between when the MoCA exam was completed, and when the neuropsychological report was created. It could be reasonably argued that the time gap affected correlations between the patient’s performance on the MoCA exam and their health data recorded in the report. The majority of the MoCA exams and neuropsychological reports were completed with a mean of 1.70 years (SD ≈ 1.38 years) of each other, apart from Patient ID 4, who had 5 years and 2 months between (Table 1). However, it should be noted that we were extracting features that are clinically relevant for *assessing* the performance on the MoCA exam by people with PD on the cognitive impairment spectrum—as noted in the ‘Final Diagnosis’ column of Table 1—rather than *determining* the presence of cognitive impairment. The purpose of presenting the three different patient profiles with a wide range of PD symptomology to each interviewee was to ensure we prompted multiple different contexts and elicited complex pattern recognition skills.

## Conclusion

Patterns in health data have previously been leveraged to differentiate the diverse presentations of PD. Imaging studies have successfully distinguished idiopathic PD from other similarly presenting degenerative disorders, such as progressive supranuclear palsy or related parkinsonisms.[45] Other research has also described the utility of a patient’s health history and physical exams in identifying clinical patterns of disease-causing pulmonary dysfunction.[46, 47] Through this lens, we can extrapolate that when patient health-related data is considered, there are discriminating disease features to be gleaned. Unfortunately, reliable and robust detection of cognitive deficits in PD remains a persistent challenge. This is particularly concerning given that PD-MCI reduces patient quality of life, is a risk factor for developing dementia, and increases the risk of early mortality.[1, 9, 13, 48] In the absence of preventative treatments for cognitive impairment in PD, and with the estimated prevalence of PD increasing globally, the need to develop robust screening exams for early detection is urgent.[49–53]

We argue that by leveraging the innate human capacity for pattern recognition,[30, 54] which is the grounded authority of the differential diagnostic process,[27] we can begin to decipher clinically relevant disease patterns within the complex symptomology of the cognitive impairment spectrum in PD. This insight could optimize cognitive exams for PD, thereby facilitating earlier identification and more timely introduction of interventions, such as exercise, to slow disease progression.[55] Extracting diagnostically meaningful patterns of cognitive impairment was the first step toward achieving that goal. The next is to quantitatively represent the extracted pattern features and determine if they exist outside of this sample data set in a large corpus of MoCA exams and patient health data.

## Supplementary Material

Supplementary Files

This is a list of supplementary files associated with this preprint. Click to download.

- Supplemental1.docx
- Supplemental2.docx

## Figures and Tables

**Table 1:** The Recorded Date of Administration for the Assessments and Final Diagnosis by Patient ID Number

Patient ID	MoCA Date	Neuropsychological Report Date	Final Diagnosis
1	4/2016	4/2014	Dementia due to PD
2	1/2016	4/2015	Mild cognitive impairment (non-amnestic)
3	10/2015	6/2014	Amnestic mild cognitive impairment
4	11/2019	9/2014	Amnestic mild cognitive impairment
5	11/2015	8/2015	Mild cognitive impairment (non-amnestic)
6	4/2021	11/2018	Dementia due to PD
7	11/2014	7/2015	Mild cognitive impairment (non-amnestic)
8	10/2019	1/2021	Mild cognitive impairment (non-amnestic)
9	6/2021	12/2019	Dementia due to PD

**Table 2:** Example Quotes with Associated Codes and Categories from Qualitative Analysis

Quote	Code	Category	Document	Important Feature(s)
*“I mean we always have their med list and medical history and things like that…”*	Use of medical background	Contextual health data for cognitive assessment	Neuropsychological report	Medication list, medical history
*“So gives me a little bit of insight, you know, years of education, what they did for a living, you know who their family is and who their support system is and that sort of thing. So, so I find those things to be…helpful to kind of put everything into context.”*	Contextual patient background	Biopsychosocial contextualization	Neuropsychological report	Years of education, occupational history, family support system
*“…when I moved down to the kind of registration part of the memory looks like it took. A few tries to get it correct, looks like they may have had some difficulty with hearing when they said faith instead of face.”*	Interpreting test behavior through sensory/context cues	Exam performance contextualization	MoCA exam	Number of tries to get MoCA memory task correct, hearing loss

**Table 3:** Patient Profile Demographics, Years of Education, MoCA and RBANS Total Scores

	Groups Compared	H Statistic	p-value	η^2^ (Effect Size)	Interpretation
Age	PD-MCI non-A, PD-MCI A, PD-Dementia	1.99	0.369	0.249	Small
Years of Edu.		2.63	0.268	0.329	Medium
MoCA		4.90	0.086	0.613	Large
RBANS		6.11	0.047^⊠^	0.764	Large

Table 3: Interpretation thresholds for η^2^: Small ≈ 0.01, Medium ≈ 0.06, Large ≥ 0.14;

⊠ p < 0.05.

**Table 4:** Clinically Meaningful Features from the MoCA exam and Neuropsychological Report

	MoCA Exam Section	Neuropsychological Report
Recurrent features in all patterns:	**Trail Task**: Evidence of errors (e.g., “Any errors”) **Cube**: Difference from ideal cube (e.g., 3D-ness)	Medication list Sleep disorder/issues Impaired independence of activities of daily living Years of education
Pattern 1:	**Trail Task**: Number of errors **Cube**: Jitter in lines, degree of skew **Clock**: Numbers not sequential or missing, no short/long hand, position of hands, contour **Language and Attention**: Lexical fluency and serial 7 (**Attention**) tasks- check lexical fluency for Alzheimer’s disease, the total number of words given in lexical fluency task	Hearing loss/aids Vision loss B12 deficiency Evidence of delirium Vascular risk factors Activity level Premorbid level of functioning
Pattern 2:	**Trail Task**: Correction of errors (extra lines) **Cube**: Jitter in lines **Clock**: Small size of numbers, evidence of correction of error (extra lines) **Attention**: Serial 7s and letter vigilance task performance (normalized by years of education), Attention test: note early stopping on letter vigilance task, delayed recall section, or lexical fluency task (**Language**) **Language**: Number of words on lexical fluency task normalized by years of education	Medication specifics (cognition-impacting drugs) Occupational exposure to pesticides Evidence of urinary tract infection Family history of dementia History of concussion(s)
Pattern 3:	**Trail Task**: Level of completion, jitter of lines **Cube**: size, shape, orientation **Clock**: Number of tries, percent complete, size of circle and numbers, contour, orientation, number spacing/order, position and length of clock hands **Memory**: Number of tries, correlate to hearing issue **Language and Attention**: Lexical fluency and serial 7 task (**Attention**) (normed by years of education) correlate with attention issues **Delayed Recall**: Categorical vs. multiple choice cue success	Medication specifics (PD medication timing, sedatives, dementia drugs) Vision loss Hearing loss/aids Arthritis Family support Vascular risk factors Cardiovascular disease indicators Premorbid level of functioning Social engagement

## Data Availability

The datasets generated and/or analyzed during the current study are available in the Zenodo repository, the interview transcripts (DOI: 10.5281/zenodo.16861070), and the Excel document describing how the patterns were derived from the interviews (DOI:10.5281/zenodo.16859473).

## Abbreviations

HIPPA: Health Insurance Portability and Accountability Act
PD: Parkinson’s Disease
PD: Dementia—Parkinson’s Disease with Major Cognitive Impairment
PD: MCI—Parkinson’s Disease with Mild (Minor) Cognitive Impairment
PD: MCI A—Parkinson’s Disease with Mild Cognitive Impairment Amnestic
PD: MCI non—A—Parkinson’s Disease with Mild (Minor) Cognitive Impairment Non—Amnestic
RBANS: Repeatable Battery for the Assessment of Neuropsychological Status
SD: Standard Deviation
